# A study on the impact of Internet use on depression among Chinese older people under the perspective of social participation

**DOI:** 10.1186/s12877-022-03359-y

**Published:** 2022-08-24

**Authors:** Hua-lei Yang, Shuo Zhang, Si-meng Cheng, Zhi-yun Li, Yuan-yang Wu, Si-qing Zhang, Jia-hao Wang, Yi-wen Tao, Yi-dan Yao, Lin Xie, Wen-jing Xiao, Xiao-qing Tang, Jing Wu, Zheng Shen, Li-li Tang

**Affiliations:** 1 School of Public Administration, Zhongnan University of Economics and Law, Wuhan, China; 2 College of Politics and Public Administration, Qingdao University, Qingdao, China; 3 School of Medicine and Health Management, Tongji Medical School, Huazhong University of Science and Technology, Wuhan, China; 4 Institution of Population and Labor Economics, University of Chinese Academy of Social Science, Beijing, China; 5 School of Economics and Management, Zhejiang A & F University, Hangzhou, China; 6 College of Chemistry and Chemical Engineering, Yantai University, Yantai, China

**Keywords:** Internet use, Older people, Depression, Social participation

## Abstract

**Purpose:**

This study aimed to evaluate the role of social participation in the relationship between internet use and depressive symptoms among Chinese older adults and investigate how the internet use interact with social participation to reduce the risk of depressive symptoms.

**Methods:**

Based on the survey from the China Health and Retirement Longitudinal Study (CHARLS) in 2018, we identified 4645 subjects and used the Ordinary Least Square method (OLS) and Propensity Score Matching method (PSM) to identify the association between Internet use and depression of older people, and further test how social participation played a role in the relationship.

**Results:**

The level of depression of older people was significantly reduced in those who using internet in China, and the effect was still robust under different identification methods. The mental health was improved when using internet because of the increase of social participation and social capital. Further, The positive effect was stronger especially in those who were female, living in rural areas, has low education attainments and were 70–79 years old.

**Conclusions:**

The popularity of internet use has a positive effect on the depressive symptoms of Chinese older adults. Effective measures were encouraged to improve the friendliness of internet for older people and promote the popularization of the Internet and older group, achieving the spiritual well-being of them in the Internet society.

## Background

Driven by the one-child policy and rapid industrialization, China’s population structure has undergone profound changes, and it has the largest scale and growth rate of older people population in the world. According to the Seventh Census in 2020, the number of people aged 60 and above was 264 million, accounting for 18.7%, of whom 191 million were aged 65 and above, accounting for 13.50% [43]. Chinese society was about to enter the stage of deep aging. The biggest problem associated with the elderly society is the elderly health problem, especially the mental health. Depression and its associated health problems such as diabetes, disability and suicide were threatening the health and quality of life of older people in China [55, 56]. The report released by the World Health Organization showed that the risk of depression peaks in middle and old age, and the older you are, the more likely you are to develop depression [62]. A meta-analysis also showed that the proportion of Chinese older people with depressive symptoms were as high as 23.6% [33]. In this context, mental disorders such as geriatric depression not only seriously affected the life quality of older people, but also increased the financial and mental stress of their families, while inevitably adding to the medical burden and resource strain on the whole community [69].

A growing body of research has been exploring risk factors for depression in old age, ranging from biological characteristics, behavioral traits, socio-economic status, family structure, living arrangements to community environment and more [2, 30]. Sociodemographic characteristics such as age, gender, education level, marital status and physical condition were all associated with individuals’ subjective well-being and affect their mental health [16]. The study of older men in Europe has shown that physical activity and moderate alcohol consumption could prevent depression in older people [5]. Other studies have also found that smoking [6], drinking [22, 25, 35], diet and sleep [38] were associated with depression. Family financial support and urban environment also affected the mental health of them [41]. Also, the “vascular depression hypothesis”, widowhood, lower socioeconomic levels, the transition from an active career to retirement, and the presence of chronic conditions such as diabetes have all been tested for their impact on depression in old age [30]. Furthermore, numerous studies have found that political factors such as the degree of democratic development [8, 17, 54] and government public services [7], and natural environmental factors such as geography and air quality [57] were all associated with subjective well-being and depression.

Among these factors, the Internet use was now attracting attention. The Internet was becoming more and more common among older people and has become an important part of daily life [34]. By June 2020, the number of Internet users in China has reached 940 million, of which the elderly users over 60 accounted for 10.3% [9].

In China, as a result of the COVID-19 crisis, people were actively complying with the epidemic prevention and control regulations, consciously reducing the number of offline meetings and gatherings, and starting to gradually move many of their daily activities online, such as online medical consultations, online work, online shopping, online chatting, etc. For older people, some of them were affected by the epidemic and began to change their perspectives on technology and the Internet, gradually accepting and learning to use the Internet to adapt to their lives now and beyond. Although the Chinese government has introduced a series of policies and measures to help and promote Internet access among older people, there were still some people who were unwilling or unable to use the Internet due to their age, lifestyle habits, literacy level and physical condition [4, 53], resulting in them being gradually left behind by the rapid development of society. So, in the Internet era, did the Internet affect the mental health of older people, and did Internet use increase well-being or depression? This has become an important social issue worldwide, and the answer to this question would go some way to helping older people adapt better to the Internet age and safeguard their mental health.

In recent years, with the gradual popularization of the Internet, the positive effect of Internet use on the health of older people has attracted the attention of many scholars. On the one hand, as an emerging technology, the Internet could provide elderly users with health care related information [61], online entertainment resources (such as videos and songs) and convenient shopping consumption [31, 64], so that they had better life experience and higher health level [39]. On the other hand, they used the Internet to maintain parent-child relations and family ties, maintain interaction with friends [69], broaden the scope of interpersonal communication and social participation [18, 67], strengthen social connection with others [14], and strengthen the social capital of them [40], which helps to reduce the risk of loneliness and depression and improve the well-being of them [11, 52].

However, the existing empirical conclusions related to Internet use and depression of older people were not consistent. Most showed that Internet use had a protective effect on depression of them in developed countries and Chinese society [12, 14, 34, 37, 55, 56, 65, 66, 69]. Some studies using randomized intervention experiments also showed that providing Internet training and expanding access to older people could significantly reduce depression and improve positive attitudes [50, 59]. However, some studies have found that there was no significant evidence that Internet use affected the mental health of older people [24, 61], and even wrong use would have an adverse impact on the mental health [21, 28, 29, 45].

Although existing studies have analyzed the impact of Internet use on health, there were few empirical tests of the specific mechanisms by which Internet use improved health and reduced the risk of depression [14, 40, 69]. As a structural social capital, social participation may be an important intermediary variable for Internet use to reduce depression of older people. Conceptually, social participation meant that people actively participated in various social activities, such as friend communication, community interaction and voluntary service, so as to form and expand social networks [13, 60]. Theoretically, in the process of participating in social activities, older people could obtain economic and emotional support, a sense of identity and self-esteem through communication and interaction with others, which was helpful to improve their mental health [1, 23]. Empirically, the existing studies showed that social participation could reduce the sense of social isolation, loneliness [10, 13, 15, 20, 26, 36, 55, 56]. Moreover, some studies have also confirmed that Internet use could improve the scope and frequency of social activities, maintain and establish social relationship networks, and expand individual social capital by strengthening contacts with friends and communities [3, 37]. Therefore, based on the previous study [65], this paper proposed that Internet use can improve social participation and strengthen social capital of older people, so as to reduce the risk of depression.

Overall, there was growing academic interest in the health effects of Internet use by older people. Based on this, using the data from the 2018 wave of the China Health And Retirement Longitudinal Study, the aim of this study was to explore the association between Internet use and depression among older people in China, and to further investigate whether the association in a socially unevenly developed Chinese scenario differs across different characteristics of older people. Also, we examined the mediating role of social participation, an overlooked mediating variable.

## Method

### Data source

**Fig. 1:**
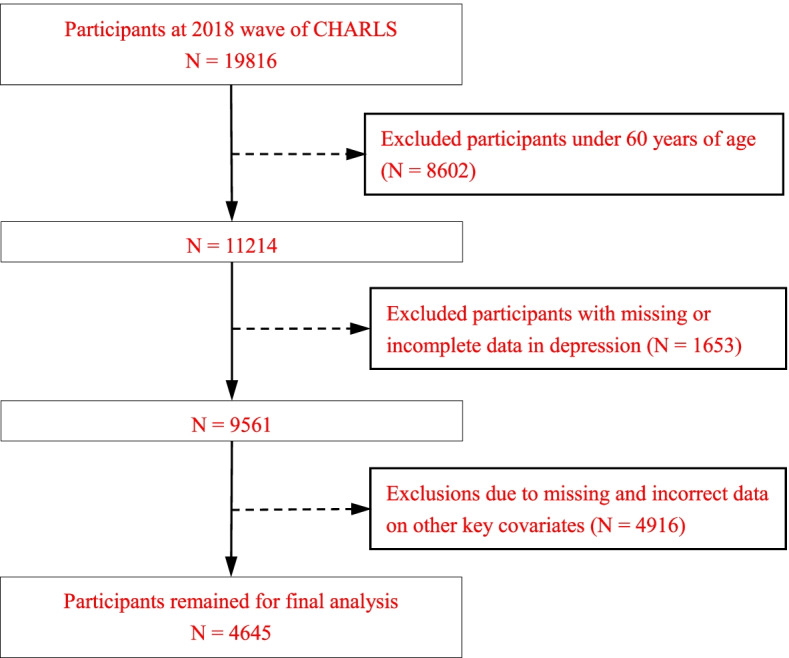
Flow chart of the study population selection process

The data from the 2018 China Health And Retirement Longitudinal Study (CHARLS) was used to conduct the analysis, the latest wave of data available at present. Details and sampling procedures of the CHARLS study were described elsewhere [68]. The survey adopted a multi-stage stratified PPS sampling process to collect a set of high-quality micro-data representing families and individuals of middle-aged and older people aged 45 and over, and the baseline wave of CHARLS was launched in 2011 to cover 28 provinces across the country. Considering the need and aim of research, older people aged 60 and above were enrolled in the study. After excluding the missing values of core variables, the final number of effective samples was 4645, among them, there were 1035 males and 3610 females. Figure 1 presented a flow chart of the study population selection process.

### Variable design

#### Dependent variable

The dependent variable of this paper was the CES-D Score of older people. The Center of Epidemiological Survey-Depression Scale (CES-D) was adopted to measure the mental health, a scale commonly used to investigate depressive symptoms in the general population [8, 48]. The scale has demonstrated high levels of internal consistency across samples and concurrent validity in both developed and developing countries [42]. Also, we confirmed the good reliability and validity of the Chinese version of CES-D with Cronbach’s alpha, which reached 0.815 in Chinese sample data of CHARLS [32]. In CHARLS, respondents were asked to answer ten questions, including two positive emotion items and eight depression items. Each item on the scale has four response options, including ‘Rarely or none of the time (<1 day)’, ‘Some or a little of the time (1–2 days)’, ‘Occasionally or a moderate amount of time (3–4 days)’, and ‘Most or all of the time (5–7 days)’ over the past week. Assign the answers under the item of depression as integers between 1 and 4 respectively, and carry out corresponding reverse assignment for the answers under the positive item. In this study, the CES-D score ranged from 10 to 40, with higher scores indicating higher degrees of depressive symptoms.

#### Independent variable

Internet use was the independent variable of this study. In the survey, respondents were asked “whether there have been Internet activities in the past month”, and we set the answer to “yes” = 1 and “no” = 0.

#### Mediating variable

This paper hypothesized that social participation was an intermediary variable for Internet use to reduce the depression level of older people, which is measured by 8 items.^1^ Then we summed up the frequency of respondents’ participation in activities and carried out standardization.^2^ The larger the value, the higher the level of social participation.

#### Control variables

It was necessary to control the impact of other confounding factors and existing relevant studies showed that personal characteristics, social environment and health-related behaviors would affect the health of older people [19, 51]. Therefore, gender, age, education level, marital status, smoking status, drinking status, residence, religious belief, political status, air quality status, medical insurance were included in the control variables. The descriptive statistics of variables were shown in Table 1.

**Table 1:** Variable description

Variable	Variable definition and assignment
Dependent variable	
CES-D score (Depressive status)	continuous variable (CES-D score range 10 to 40) the higher the score, the more serious the depression.
Independent variable	
Internet use	yes = 1, no = 0
Control variables	
Gender	man = 1, woman = 0
Age	actual age at the time of interview survey year - respondent’s year of birth
Education level	primary school and below =1, junior high school = 2 senior high school = 3, higher education = 4
Marital status	married = 1, separated, single, divorced or widowed = 0
Smoking status	yes = 1, no = 0
Drinking status	yes = 1, no = 0
Residence	urban = 1, rural = 0
Religious belief	yes = 1, no = 0
Political status	the Chinese Communist party member = 1, other = 0
Air quality status	completely satisfied = 5, very satisfied = 4, somewhat satistied = 3, not very satisfied = 2, not at all satisfied = 1
Medical insurance	covered medical insurance = 1, without medical insurance = 0
Mediating variable	
Social capital	Standardized social capital index

### Methods

#### Ordinary least squares (OLS)

Given that the CES-D score used in this study was continuous variable, we used the Ordinary Least Sqaure (OLS) model to examine the relationship. The model was constructed as followed:

1 $${Score}_{it}={\alpha}_1+{\beta}_1{Internet}_{it}+{\gamma}_1{X}_{it}+{\delta}_{it}$$

Where *Score*_*it*_ represented the CES-D score of older people, *Internet*_*it*_ represented the use of Internet, *X*_*it*_ was other control variables affecting the depression, and *δ*_*it*_ was a random error term. *α*_1_ denoted the intercept term. *β*_1_ and *γ*_1_ represented the regression coefficient for the corresponding variable.

#### Propensity score matching model (PSM)

Theoretically, whether older people used the Internet was a self-selection behavior, so there may be a selective bias. The Propensity Score Matching method was used to test the robustness to determine the net impact of Internet use on depression. The setting mode was as followed:

2 $${y}_i=\left(1-{D}_i\right){y}_{0i}+{D}_i{y}_{1i}={y}_{0i}+\left({y}_{1i}-{y}_{0i}\right){D}_i$$

3 $$ATT=E\left[{y}_{1i}-{y}_{0i}|{D}_i=1,P(X)\right]=E\left[{y}_{1i}|{D}_i=1,P(X)\right]-E\left[{y}_{0i}|{D}_i=1,P(X)\right]$$

Where*y*_1*i*_represented the score of older people using the Internet and *y*_0*i*_ represented the score of older people who did not use the Internet; The processing variable *D*_*i*_ = {0, 1} indicated whether the respondent used the Internet, where 1 represented the treatment group and 0 represented the control group.

#### Mediating effect model

In order to explore the mechanism of the impact of Internet use on depression of older people, the mediating effect model was constructed with reference to the method of Wen et al. [58]. The models were set as followed:

4 $$Social\hbox{-} {capital}_{it}={\alpha}_2+{\beta}_2{Internet}_{it}+{\gamma}_2{X}_{it}+{\phi}_{it}$$

5 $${Score}_{it}={\alpha}_3+{\beta}_3{Internet}_{it}+{\lambda}_1 Social\hbox{-} {capital}_{it}+{\gamma}_3{X}_{it}+{\varphi}_{it}$$

Where *α*_2_represented the influence coefficient of Internet use on mediating variable, and *α*_3_ represented the influence coefficient of Internet use on CES-D score of older people after adding mediating variable.

## Results

### Descriptive analysis

Table 2 showed the basic descriptive statistics of older people using and not using the Internet. The average CES-D score of older people who used the Internet was 15.637, which was lower than the overall average and the average of older people not using the Internet, indicating that their mental health was better. Only 7% of respondents said they would use the Internet for relevant activities. The average age of whole sample was about 68.168 years old, among which the age of user was relatively low. Older people who used the Internet had a higher level of education, and 72.2% of user lived in cities and towns, while most of non-user lived in rural areas,

**Table 2:** Basic descriptive statistics

Varname	Mean			Mean-Diff	T value
	Total (*N* = 4645)	Not using the Internet (*N* = 4312)	Using the Internet (*N* = 333)		
CES-D score	19.268	19.548	15.637	3.912^***^	10.013
Gender	0.223	0.215	0.318	−0.103^***^	−4.354
Age	68.168	68.333	66.024	2.309^***^	6.607
Education level	1.374	1.294	2.408	−1.114^***^	−27.448
Marital status	0.785	0.780	0.841	−0.060^***^	−2.588
Smoking status	0.072	0.068	0.123	−0.055^***^	−3.781
Drinking status	0.205	0.192	0.366	−0.174^***^	−7.632
Residence	0.315	0.278	0.790	−0.511^***^	−20.188
Religious belief	0.131	0.133	0.108	0.025	1.279
Political status	0.085	0.068	0.297	−0.229^***^	−14.776
Air quality status	3.192	3.216	2.883	0.333^***^	7.143
Medical insurance	0.974	0.972	0.994	−0.022^**^	−2.400
Social capital	0.052	−0.013	0.904	−0.918^***^	−15.933

*Note*: ^*^ *p* < 0.10, ^**^ *p* < 0.05, ^***^ *p* < 0.01

### Regression results

The regression results for associations between Internet use and depressive symptoms were presented in Table 3. It could be found that Internet use of older people reduced CES-D score and improved their depression status without adding control variables in Model 1. After adding control variables, the above conclusion was still valid, as shown in Model 2. Older people using the Internet had 1.800 points lower scores of depressive symptoms than non-user (*P* < 0.01). In addition, most control variables affected the depression status of them, gender, marital status, smoking and drinking status and air quality status were significantly negatively correlated with CES-D score (*P* < 0.01 for all).

**Table 3:** Baseline regression results

Variable	Model 1	Model 2
Internet use	−3.912^***^	− 1.800^***^
	(0.294)	(0.332)
Gender		−1.715^***^
		(0.254)
Age		−0.005
		(0.017)
Education level		−0.913^***^
		(0.138)
Marital status		−1.282^***^
		(0.260)
Smoking status		1.119^***^
		(0.372)
Drinking status		−0.995^***^
		(0.238)
Residence		−2.146^***^
		(0.230)
Religious belief		−0.773^***^
		(0.287)
Political status		−0.458
		(0.348)
Air quality status		−1.312^***^
		(0.126)
Medical insurance		0.253
		(0.563)
*N*	4645	4645
*R*^2^	0.021	0.103

*Note:* Standard errors in brackets, ^*^ *p* < 0.1, ^**^ *p* < 0.05, ^***^ *p* < 0.01

### Propensity score matching analysis

In order to control the selectivity bias of whether older people used the Internet, this paper further used the PSM method to estimate the average processing effect of Internet use on CES-D score. The balance test results of samples were shown in Table 4. The standard deviation of variables after matching was greatly reduced, and there was no significant difference between treatment group and control group. Therefore, the systematic differences of variables could be eliminated to a great extent.

**Table 4:** Balance test

Variable	Mean		%bias	T-test		V(T)/V(C)
	Treated	Control		t	P> | t |	
Gender	0.312	0.312	0.00	0.00	1.000	.
Age	66.095	66.422	−5.60	−0.75	0.453	0.88
Education level	2.379	2.388	−1.00	−0.11	0.916	0.78^*^
Marital status	0.838	0.872	−8.60	−1.22	0.223	.
Smoking status	0.122	0.104	6.30	0.74	0.460	
Drinking status	0.358	0.321	8.30	0.99	0.322	
Residence	0.786	0.807	−5.00	−0.68	0.497	
Religious belief	0.107	0.104	0.90	0.13	0.899	
Political status	0.284	0.300	−4.10	−0.43	0.668	
Air quality status	2.899	2.884	1.90	0.25	0.801	0.98
Medical insurance	0.994	0.994	0.00	0.00	1.000	.

*Note:* ^*^ *p* < 0.1, ^**^ *p* < 0.05, ^***^ *p* < 0.01

Table 5 reported the average processing effect estimation results of five methods: K-nearest neighbor matching, radius matching, K-nearest neighbor matching in caliper, kernel function matching and local linear regression matching. It could be found that the above conclusion was still valid. These results revealed a strong protective role of Internet use against depressive symptoms for Chinese elderly.

**Table 5:** PSM estimation results

Matching method	Treatment group	Control group	ATT	Bootstrap standard error	T value
K-nearest neighbor matching	15.661	17.439	−1.779^***^	0.446	−3.40
Radius matching	15.666	17.432	−1.766^***^	0.363	−4.51
K-nearest neighbor matching in caliper	15.666	17.456	−1.790^***^	0.514	−3.42
Kernel matching	15.661	17.536	−1.875^***^	0.322	−5.05
Local linear regression matching	15.661	17.519	−1.858^***^	0.365	−3.55

*Note*: ^*^ *p* < 0.10, ^**^ *p* < 0.05, ^***^ *p* < 0.01; K = 1; standard error after matching is obtained by bootstrap method

### Heterogeneity analysis

The above research showed that Internet use significantly improved depression of older people, but this effect may be different in different groups, as shown in Table 6. Models 3–5 showed that the conclusion that using the Internet improved depression was applicable to older people under the age of 80, and users among those aged 70–79 had 2.402 points lower scores than non-users (*P* < 0.01). As shown in Models 6 and 7, whether older women or men, using the Internet significantly reduced the CES-D score (P < 0.01 for all), especially for women group. Internet use had a greater impact on older people with lower education level. Model 8 showed that among primary school and below group, using the Internet could reduce the score by 2.316 points (*P* < 0.01). Results of Models 11 and 12 showed that Internet use significantly reduced the CES-D scores of urban and rural older people (P < 0.01 for all), and had a greater impact on those living in rural, indicating that Internet use was more helpful to improve the mental health of rural older people.

**Table 6:** Estimation results of heterogeneity analysis

Variable	Age			Gender	
	Model 3	Model 4	Model 5	Model 6	Model 7
	Age = 60–69	Age = 70–79	Age ≥ 80	Man	Woman
Internet use	−1.606^***^	−2.402^***^	0.607	−1.552^***^	− 1.837^***^
	(0.391)	(0.711)	(1.823)	(0.515)	(0.419)
Control variable	Yes	Yes	Yes	Yes	Yes
*N*	3020	1345	280	1035	3610
*R*^2^	0.094	0.126	0.131	0.089	0.088
Variable	Education			Residence	
	Model 8	Model 9	Model 10	Model 11	Model 12
	Primary school and below	Junior middle school	High school and above	Urban	Rural
Internet use	−2.316^***^	−1.453^**^	−1.687^***^	−1.569^***^	−2.655^***^
	(0.619)	(0.574)	(0.543)	(0.371)	(0.728)
Control variable	Yes	Yes	Yes	Yes	Yes
*N*	3548	648	449	1463	3182
*R*^2^	0.071	0.092	0.076	0.081	0.075

*Note*: ^*^ *p* < 0.10, ^**^ *p* < 0.05, ^***^ *p* < 0.01; standard errors are reported in parentheses

### Mechanism analysis

In order to identify the mediating effect of social participation, we used the statistical method of Wen et al. [58] to test the mediating effect of social participation. The specific results were shown in Table 7. Model 14 showed that there was a significant positively association between Internet use and social participation of older people (coefficient = 0.638, P < 0.01). After adding intermediary variables, the association between Internet use and CES-D score of older people was still significant (P < 0.01), and the coefficient was reduced from 1.800 to 1.450. These results indicated that social participation played an intermediary role in Internet use to reduce depression and it was seen that the indirect effect accounted for 19.46% of the total effect through calculation.

**Table 7:** Estimation results of mediation mechanism analysis

Variable	Model 13	Model 14	Model 15
	CES-D score	Social capital	CES-D score
Internet use	−1.800^***^	0.638^***^	−1.450^***^
	(0.332)	(0.087)	(0.333)
Social capital			−0.549^***^
			(0.090)
Control variable	Yes	Yes	Yes
*N*	4645	4645	4645
*R*^2^	0.103	0.081	0.109

*Note*: ^*^ *p* < 0.10, ^**^ *p* < 0.05, ^***^ *p* < 0.01; standard errors are reported in parentheses

## Discussion

With the worldwide population aging, the mental health of older people has gained an increasing focus. Based on data from the 2018 China Health And Retirement Longitudinal Study, we sought to explore the association between Internet use and depression in older people and to examine the mediating role of social participation. Consistent with most existing research conclusions [14, 34, 66], this study confirmed that Internet use had a protective effect on depression of older people in China. Studies have explained the possible mechanism from two aspects. On the one hand, compared with traditional media, the Internet could provide older people with rich health care information, online entertainment resources, etc., so that they had a better life experience, which was conducive to improving their mental health [61, 63]. On the other hand, with the growth of age, especially after retirement, their original work-related interpersonal relationships disappeared, resulting in the disconnection between old people and society. As an emerging technology, the Internet could help the them maintain and establish social relations, expand the scope of social participation and accumulate social capital across time and space, so as to alleviate the sense of isolation [14, 34].

In fact, with the help of online platforms, older people could actively participate in social activities and strengthen their offline social participation by more convenient access to all kinds of information, which helped to reduce the risk of depression. In addition, Zhu et al. [69] found that the structural social capital characterized by “interaction with friends” did not show a significant mediating effect. Lyu and Sun [40] used the “gift expenditure” of the past year to describe the structural social capital, which had a significant mediating effect on Internet use to improve self-rated health of older people, but the indirect effect accounted for only 2.45%. This may be because in the Chinese society with obvious characteristics of “differential order pattern”, the social capital described by “communicating with friends” and “gift expenditure” was limited to the close acquaintance relationship network, which could not reflect the positive role of Internet use in expanding social communication and participation [3]. With the growth of age, the circle of acquaintances was shrinking, and the Internet can help them participate in various social activities, enhance community connection and establish new social networks [44, 46]. Therefore, this paper used eight types of social activities [36] including informal social participation (such as friend interaction, providing informal help to family and neighbors) and formal social participation (such as community organization activities, volunteer services, etc.) to measure the comprehensive level of social participation of older people, and it was found that active and wider social participation was one of the important channels for Internet use to reduce the risk of depression in older people.

Internet use had a stronger correlation with mental health among middle and lower aged older adult [65, 69]. Relatively older adults could have more learning and technological barriers to Internet use. At the same time, due to the decline of physical function and limited activity ability, they may not be able to participate in community activities. So, the protective effect of Internet use on depression of them was very limited [47, 67]. On the contrary, younger older adults could use the information accessibility of the Internet to maintain an interactive network with friends and even compensate for the network of colleagues and related activity participation lost through retirement [49], thus gaining a sense of identity and psychological belonging, which helped to maintain the mental health of them. From the perspective of education level, older people with lower education level would obtain more health effects in the process of Internet use [27, 39]. This may be because older people groups with different education levels had different social resources and social networks, and the Internet had a greater impact on the lifestyle of those with lower education levels. For example, the emergence of short video applications would strengthen the contact and interaction of this group, expand the social network and improve the level of social adaptation [27]. Compared with older people living in urban, the rural elderly benefited more from the Internet, which was consistent with Liao et al. [34] and Zhu et al. [69]. There may be the following two reasons: firstly, there were great differences in medical resources and health information between them and there was a lack of medical resources in rural areas. As one of the sources of health information, the Internet promoted the health of the rural elderly; Secondly, compared with urban residents, farmers had fewer channels for social activities, so online platforms could effectively make up for the lack of social participation of them to reduce the risk of depression.

## Conclusions

In summary, this paper empirically demonstrated the positive impact of Internet use on depressive symptoms among older people in China and the role of social participation in this relationship. Interventions should be developed to help older individuals with depressive symptoms based on the findings of this paper.

Firstly, make full use of the Internet to effectively prevent and treat mental disorders, such as, depression of older people. The Internet accessibility and convenience of older people should be further promoted. In some rural areas, the Chinese elderly, discarded by digital society, generally have poor access to the Internet. However, it was mentioned that modern technology was mainly aimed at young people. There were significant differences between the elderly and young people in terms of physical function and psychological cognition, and the product design was not applicable to the elderly, such as disordered page layout, small web page font, vulgar information content, etc., which brought unfriendly experience to the elderly and damaged their physical and mental health. Therefore, the existing equipment and applications need to be gradually incorporated into the friendly design for the elderly users, especially considering the decline of the elderly users’ visual and auditory abilities, and carrying out adaptation in the aspects of voice, character recognition, font size, etc., so as to improve the experience of the elderly in using the Internet. Besides, other measures were also worth advocating, such as strengthening the review and supervision of relevant contents for the elderly in the Internet, using big data to more accurately tap the potential needs of the elderly, and providing targeted services and commodities for the elderly group.

Secondly, built Internet platform to realize the social participation of the elderly. In platform, the elderly actively carry out online and offline activities suitable for older people, provide special activity venues in the community, and improve the level of social integration of them, so as to maintain and expand the social network and reduce the risk of depression of them.

Finally, due to the group differences of the depression of older people, interventions should be implemented for different types of elderly groups. For example, for older people with poor learning ability, we should help them accept and use the Internet by encouraging family guidance and social workers’ participation, so as to finally bridge the “digital divide”; In view of differences between urban and rural areas, government departments should strengthen the inclusive construction of Internet infrastructure, gradually improve the rural Internet penetration rate, and reduce the differences in Internet use, to promote the healthy and balanced development of all kinds of elderly people, and finally improve the well-being of all older people in the Internet era.

There were several major limitations to be noted in this study. First, we recognized the cross-sectional nature of data in the study, coupled with the fact that this was an observational study, so any conclusions about predictions could only be understood in a statistical sense and did not provide evidence for causality. Second, in order to gain a fuller understanding of the role of social capital in health, more direct and extensive measures of bridging and linking social networks were necessary. However, there was not much more available information in the existing data. Future research was definitely needed to gather more information in this area and to further elucidate how different forms of social capital moderate the relationship between Internet use and health.

## Data Availability

The datasets generated and/or analyzed during the current study are available in the China Health and Retirement Longitudinal Study repository, http://charls.pku.edu.cn/pages/data/111/zh-cn.html

## Footnotes

1 Specifically, in the survey, respondents reported whether they had participated in the following social activities in the past month: (a) visiting and communicating with friends; (b) playing mahjong, chess and cards, going to community activity rooms and other recreational activities; (c) provide help to relatives, friends or neighbors who do not live with you; (d) dancing, fitness and qigong exercises; (e) participate in community organization activities; (f) volunteer activities or charitable activities; (g) take care of patients or persons with disabilities who do not live with you; (h) go to school or attend training courses.

2 Standardization method: individual value minus mean and then divided by standard deviation.
